# Acute pseudo-septic arthritis following viscosuplementation of the knee

**Published:** 2012-06-23

**Authors:** Zineb Idrissi, Karima Benbouazza, Maryam Fourtassi, Hanae Raissouni, Meriem El Aadmi, Fatima Zanat, Najia Hajjaj-Hassouni

**Affiliations:** 1 Rheumatology Department, El Ayachi Hospital, Mohammed the Vth University, Rabat, Morocco; 2 Physical medicine and rehabilitation Department, El Ayachi Hospital, Mohammed the Vth University, Rabat, Morocco

**Keywords:** Pseudoseptic arthritis, Hyaluronic acid, complicaton, viscosupplementation, knee

## Abstract

A 70-year-old woman with a history of medial femoro-tibial compartment of knee osteoarthritis was admitted for acute arthritis six days after a second intra-articular injection of Hyaluronic acid. The joint fluid was inflammatory, with no crystals, and laboratory tests showed marked inflammation leading to antibiotic treatment for suspected septic arthritis. The persistent symptoms and negative results of joint fluid and blood cultures led to discontinuation of the antibiotic therapy after 10 days. Anti-inflammatory with rehabilitation therapy of the knee relieved the symptoms, and the patient was discharged home 3 weeks after her admission. Aseptic arthritis induced by repeated Hyaluronic acid injection is the most likely diagnosis. Physicians should be conscious of this extremely severe complication.

## Introduction

Intra-articular hyaluronic acid (HA) injection is one of several other treatments available for knee osteoarthritis. It provides both articular cartilage and synovial membrane with visco-elastic protection [1]. As a therapeutic agent, HA has no pharmacological nor immunological effects but it can improve the rheological properties of the synovial liquid resulting in pain alleviation and joint-mobility improvement [1, 2]. Very few cases of aseptic arthritis complicating intra-articular injection of HA were described [3]. We report a new case of pseudo-septic acute mono-arthritis of the knee revealed six days after an intra-articular injection of HA.

## Patient and case report

A 70-year-old woman with medial femoro-tibial gonarthrosis underwent two intra-articular HA injections (Osténil^®^) because of the persistence of pain while walking and restricted ability to walk. Both injections were performed under aseptic conditions. Six days after the second injection, she presented with acute mono-arthritis, major functional impotence and fever. Joint fluid analysis showed 12,000 cells per mm^3^ with 83% of neutrophils and 17% of lymphocytes. A knee aspiration was performed to evaluate the possibility of a joint infection. The examination of synovial fluid under phase contrast and polarizing microscope showed no crystals, and culture on standard media was negative. The biological assessment showed an erythrocyte sedimentation rate at 80mm, C Reactive protein at 160mg/l, and white blood cell count was 10,000 elements per mm^3^. In the light of these findings, acute septic arthritis was considered to be the most likely diagnosis, and probabilistic intra-venous antibiotic therapy, with Getamycine 160 mg per day and flucloxacilline 2g every 6 hours, was started. The persistent symptoms and negative results of joint fluid and blood cultures did not plead in favour of a septic origin. In front of this beam of arguments, antibiotic therapy was stopped after 10 days, non-steroidal anti-inflammatory drugs were administered and rehabilitation therapy of the knee was started. The clinical and laboratory test abnormalities were normalized within two weeks.

## Discussion

Viscosupplementation by intra-articular injection of HA seems to be practically risk free [3–5]. The most common adverse event associated with their use is an inflammatory reaction or a flare at the injection site [5].The occurrence of non microcrystalline aseptic arthritis after injection of HA was rarely described [3, 4].

All the cases occurred after injection of hylane GF-20 (Synvisc^®^) which is a polymer of hyaluronate [3] and only one case with the sodium hyaluronan (Ostenil^®^) [5]. The delay of appearance of arthritis is generally short, lower than 72 hours [5]. The joint fluid is always inflammatory. The evolution is quickly satisfactory thanks to non steroidal anti-inflammatory drugs or corticoids allowing a complete recovery without relapse within a few days to three weeks maximum [3–5].

Our case has several particularities. The onset delay was six days versus 72 hours in other reported cases. On the other hand, our patient presented with a severe arthritis mimicking an acute septic arthritis after using (Ostenil^®^) which is known to produce no allergenic reactions [4]. The quickly favourable clinical and biological evolution under only symptomatic treatment allowed correcting the diagnosis. However, at the onset of arthritis after viscosupplementation, infectious complications should always be first and foremost, ruled out by realising a systematic joint aspiration with cytological and bacteriological analysis of the synovial fluid.

The mechanism of acute arthritis after HA injection is not clearly understood. Several hypotheses have been put forth to explain the physiopathology of these arthritis. The fact that they never occur after the first injection suggests the possibility of immune sensitization phenomenon occurring after HA accumulation [6]. The role of pro-inflammatory cytokines which could be triggered by some HA degradation products is another hypothesis to be considered [7]. It should be noted that HA interacts with its receptor CD44, which is involved in leucocytes migration and recruitment during inflammation. Moreover, CD44 is over expressed in osteoarthritis [9]. Finally, it is also possible that this arthritis was caused by some particles not removed during the purification of this product.

## Conclusion

In our case, the severity of clinical presentation, mimicking septic arthritis and the most likely accountability of HA deserve to be reported. We think that both doctors and patients should be aware of this uncommon but sometimes confusing side effect of viscosuplementation with hyaluronic acid.
